# High‐Resolution Angle‐Resolved Photoelectron Spectroscopy of ${\mathrm{Cu}}_{5}^{-}$ Clusters

**DOI:** 10.1002/cphc.70605

**Published:** 2026-10-25

**Authors:** Lukas Weise, Steve Takouan Tchounga, Bernd von Issendorff

**Affiliations:** ^1^ Physikalisches Institut Fakultät für Physik und Mathematik Universität Freiburg Freiburg im Breisgau Germany; ^2^ FMF Universität Freiburg Freiburg im Breisgau Germany

**Keywords:** angular distributions, cluster anions, copper, density functional theory, electronic structure, photoelectron spectroscopy, vibrational structure

## Abstract

Angle‐resolved photoelectron spectroscopy (ARPES) has been used to probe the electronic and vibrational structure of the ${\mathrm{Cu}}_{5}^{-}$ cluster. High‐resolution spectra recorded near the detachment threshold reveal well‐resolved vibrational progressions, enabling a precise determination of the adiabatic electron affinity (EA = 1.8947(12) eV). Measured vibrational frequencies are in good agreement with density‐functional‐theory predictions, allowing the assignment of the observed modes. The anisotropy parameter β of the photoelectron angular distribution from the highest occupied molecular orbital follows the universal momentum‐dependent behaviour recently predicted. In some cases β exhibits a dependence on the final vibrational excitation of the cluster which deviates from the prediction, revealing a possible influence of autoionizing excited states.

## Graphical abstract

Photoemission from a cluster anion often leaves the neutral cluster in a vibrationally excited state, which alters both the kinetic energy of the emitted electron and its angular distribution. Measuring these distributions therefore not only characterises the neutral cluster, but also the electron/ion coupling during photoemission.

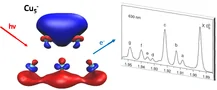

## 1 Introduction

Copper is an interesting material. Like the other coinage metals and the alkaline metals, it is monovalent, leading to a free‐electron‐gas‐like electronic system, at least close to the Fermi energy. This is one of the reasons why copper was one of the first metals studied in the form of clusters by photoelectron spectroscopy [1, 2], which provided evidence for the existence of an electron shell structure, a clear sign of free‐electron behaviour. Nevertheless, the electronic structure of copper is more complex due to the existence of the 3d‐band starting only about 2 eV below the Fermi energy. This leads to appreciable hybridisation between the localised d‐band and the free‐electron‐like s/p band, which significantly changes the interatomic bonding and could be expected to also perturb the electron shell structure [3]. It was therefore somewhat surprising that sodium and copper clusters exhibit rather similar photoelectron spectra for cluster anions with 20–40 atoms [3], calling for further studies. As smaller clusters can be characterised in much more detail both by experiment and theory, some smaller sizes should be studied as comprehensively as possible. For the study reported here we chose ${\mathrm{Cu}}_{5}^{-}$, which is small, but already more complex than a dimer or trimer.

Copper cluster anions and ${\mathrm{Cu}}_{5}^{-}$ in particular have been studied by anion photoelectron spectroscopy since the late 1980s [1, 4, 6]. Lineberger and coworkers [4] reported a vertical detachment energy (VDE) of (1.94±0.05) eV and a detachment onset of (1.82±0.05) eV; the spectra obtained in the other studies also indicate VDE values close to 2 eV, but no explicit values were given. In none of these measurements, the vibrational structure was resolved. Multiple density‐functional‐theory investigations have consistently identified a planar trapezoidal $C_{2v}$ geometry as the ground state for both ${\mathrm{Cu}}_{5}^{-}$ and neutral ${\mathrm{Cu}}_{5}$ [7, 8, 9, 10, 11, 12, 13]. This structural assignment was independently confirmed by temperature‐dependent femtosecond NeNePo spectroscopy on neutral ${\mathrm{Cu}}_{5}$ prepared by photodetachment of cryogenically cooled ${\mathrm{Cu}}_{5}^{-}$, which yielded the frequencies of the four totally symmetric $a_{1}$ modes of the cluster [14]. Other studies on small copper clusters have looked at the infrared absorption of cluster cations [15], the optical absorption of neutral clusters in a Ne matrix [16], or the decay mechanisms of highly excited cluster anions in electrostatic ion traps [17, 18].

Here we first present photoelectron spectra measured at different wavelengths on cryogenically cooled ${\mathrm{Cu}}_{5}^{-}$, which yield precise values for the adiabatic electron affinity and vibrational frequencies of the neutral ${\mathrm{Cu}}_{5}$ cluster. The second part of the report is devoted to angle‐resolved photoelectron spectroscopy. The angular distributions of the photoelectrons turn out to be in surprisingly good agreement with the predictions of a simple model for spherical free‐electron systems [19]. Nevertheless, the dependence of the angular distribution on vibrational excitation exhibits some interesting deviations, which hint at the influence of electronically excited states of the anion.

## 2 Experiment

Copper clusters were produced in a magnetron sputter gas aggregation source using a liquid‐nitrogen‐cooled 4:1 He/Ar mixture at ≈0.2mbar. Charged clusters were guided by radio‐frequency (RF) ion guides into a dodecapole RF ion trap cooled to 7K by a helium refrigerator, where they thermalised through collisions with He buffer gas. From the trap, the clusters were extracted into a double‐reflectron time‐of‐flight mass spectrometer, where a given size was selected by a Bradbury–Nielsen mass gate positioned at the focus point of the first reflector. The size‐selected cluster ion packages were then refocused by the second reflector into the interaction region of a coaxial velocity map imaging spectrometer (VMI). Here, they were irradiated by 4 ns laser pulses from an Ekspla NT342B system, continuously tunable from 225 to 2300 nm, with a polarisation aligned perpendicular to the spectrometer axis. The energy calibration of the laser was tested by performing opto‐galvanic measurements on neon and iron gas discharges. The VMI spectrometer is based on an improved design described in detail by Bartels [20]. Photoelectrons are detected by a 40 mm diameter multichannel plate with a phosphor screen, the images of which are recorded by a CCD camera. Camera frames were analysed online with a centroid algorithm identifying electron impact positions. The projected photoelectron distributions were Abel‐inverted with the MEVELER algorithm [21] after a circularisation step [22] that removes stray‐field‐induced deformations. Energy calibration was performed using the well‐known photoelectron spectrum of Pt anions.

## 3 Results and Discussion

Angle‐resolved photoelectron spectra of ${\mathrm{Cu}}_{5}^{-}$ were recorded at laser wavelengths between 250 and 650 nm, with the measurements at the longest wavelengths yielding the best resolution [23]. Representative angle‐integrated spectra obtained at 325, 553, and 630 nm are shown in Figure 1. The spectrum measured at the highest photon energy reveals three electronic bands, corresponding to detaching an electron from the highest occupied orbital or the next two lower lying ones, resulting in the ground or the first two excited states of neutral ${\mathrm{Cu}}_{5}$. In the spectrum measured at 553 nm only the X‐band can be seen, corresponding to the electronic ground state of ${\mathrm{Cu}}_{5}$; here vibrational structure is already visible. In the spectrum measured at 630 nm, several vibrational modes can be distinguished—the values of their frequencies are given in Table 1. The vibrational structure also allows one to identify the 0‐0 transition and therefore the adiabatic electron affinity, for which a value of EA = 1.8947(12) eV is obtained, in good agreement with earlier lower‐resolution studies [4, 5, 6].

**FIGURE 1:**
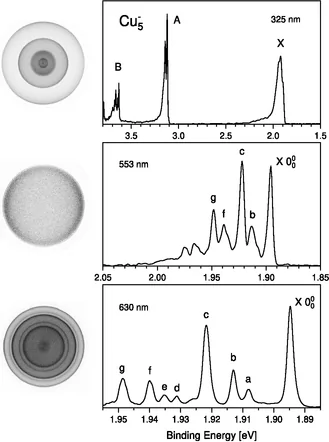
Photoelectron images and resulting photoelectron spectra of ${\mathrm{Cu}}_{5}^{-}$ recorded at different wavelengths.

**TABLE 1:** Relative energies of the features observed in the photoelectron spectrum (Figure 1) and their assignment in terms of the vibrational modes given in Table 2. Values from Ref. [14] are given for comparison.

Peak	Frequency	Assignment	**Frequency [** 14 **]**
a	109(3) cm^−1^	$\nu_{4}$	108 ${\mathrm{cm}}^{-1}$
b	147(4) cm^−1^	$\nu_{3}$	148 ${\mathrm{cm}}^{-1}$
c	218(5) cm^−1^	$\nu_{1/2}$	216/222 ${\mathrm{cm}}^{-1}$
d	295(6) cm^−1^	2× $\nu_{3}$	
e	327(6) cm^−1^	$\nu_{4}$ + $\nu_{1/2}$	
f	364(7) cm^−1^	$\nu_{3}$ + $\nu_{1/2}$	
g	434(8) cm^−1^	2× $\nu_{1/2}$	

We obtain vibrational frequencies very close to those measured by Han et al. in their NeNePo experiment [14]; their values are also given in Table 1. There is one difference: while they could resolve four different modes, we could only resolve three: the two modes with frequencies of 222 and 216 cm^−1^ are too close together to be distinguishable in the spectrum shown in Figure 1.

In order to assign the observed modes, we have performed DFT calculations with the program Turbomole [24], employing the PBE0 functional with a def2‐TZVP basis set. Both ${\mathrm{Cu}}_{5}^{-}$ and ${\mathrm{Cu}}_{5}$ optimise to a planar trapezoidal structure of C_2*v*_ symmetry, as was found many times before [7, 8, 9, 10, 11, 12, 13]. Vibrational frequencies and the corresponding displacement patterns were calculated in the harmonic approximation. Upon photoemission, normally only totally symmetric vibrations can be excited. ${\mathrm{Cu}}_{5}$ has four of these, the calculated displacement patterns and frequencies of which are presented in Table 2. For comparison, the frequencies calculated by Han et al. are given as well, who did DFT calculations with the same functional but a slightly better basis set (def2‐TZVPP) and a different program (Gaussian 16) [14]. The results are practically identical. The calculated frequencies nevertheless deviate from the observed ones—these are overestimated by 7% on average. Here, certainly, higher level calculations would be helpful. The same is true for the electron affinity—for the adiabatic electron affinity, we calculate a value of 1.59 eV, which is too low by about 0.3 eV. Interestingly, in a recent report, Li et al. obtained a value of 2.17 eV, using the B3P86 functional and a LANL2DZ basis set [12]—again off by almost 0.3 eV, but too high in this case. In the Supporting Information, we present additional results for a number of popular functionals—the results scatter strongly. This demonstrates that for a reliable calculation of electron affinities, higher level calculations are mandatory.

**TABLE 2:** Simulated displacement patterns and frequencies of the four totally symmetric vibrational modes of ${\mathrm{Cu}}_{5}$. Values from Ref. [14] are given for comparison.

Mode	Pattern	Frequency	**Lit. [** 14 **]**
$\nu_{1}$	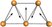	211.9 cm^−1^	213 cm^−1^
$\nu_{2}$	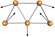	201.0 cm^−1^	201 cm^−1^
$\nu_{3}$	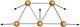	138.1 cm^−1^	139 cm^−1^
$\nu_{4}$	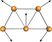	100.2 cm^−1^	100 cm^−1^

It is instructive to look at how the vibrations get excited by photoemission. In Figure 2, the highest occupied orbital (HOMO) of ${\mathrm{Cu}}_{5}^{-}$ is shown, as well as the structure of the cluster with the bond lengths of both the anion and the neutral indicated. The HOMO clearly has binding character along the upper and lower row of atoms in the trapezoidal pentamer and antibinding character between the rows. Detachment of the electron from this orbital should therefore lead to an increase in the bond lengths within the two rows and a decrease in the lengths of the bonds between the rows. Figure 2b shows that this is indeed the case; the most pronounced change is the shortening of the bond between the outer atoms of the upper and lower row, but one can also observe a lengthening of the bonds of the upper and lower row. This is a deformation pattern most clearly shown by mode $\nu_{2}$—this mode should therefore be most strongly excited. Indeed this is reflected in the Franck–Condon‐factors calculated by Han et al. [14]; they obtain very similar factors for the modes $\nu_{1}$, $\nu_{3}$, and $\nu_{4}$, and a factor about twice as large for $\nu_{2}$. Our measured spectrum is in very good agreement with this prediction: the prominent features are peaks c and g, corresponding to single and double excitation of mode $\nu_{2}$ (and mode $\nu_{1}$). It is interesting that Han et al. obtain a completely different intensity distribution of the vibrational modes in their NeNePo spectra, although the vibrational excitation of the neutral cluster after the photoemission induced by the pump pulse in principle should be exactly the same as in our case (provided that only one photon is absorbed, which might not be the case). Obviously the efficiency of the probe step (multi‐photon ionisation to produce a cationic cluster, followed by fragmentation) depends much more on the nuclear motion in the $\nu_{4}$ mode than on that in the other modes, due to which this mode dominates the NeNePo spectrum, despite being only fairly weakly excited. So, one could state that the NeNePo method reaches an impressive resolution and is therefore well suited to test predictions of vibrational frequencies. It is, however, maybe less suited to judge the deformation caused by the photodetachment (Figure 2).

**FIGURE 2:**
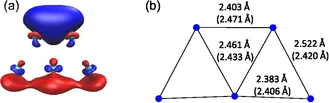
DFT simulation results: (a) HOMO of ${\mathrm{Cu}}_{5}^{-}$, (b) bond lengths of ${\mathrm{Cu}}_{5}^{-}$ (values for ${\mathrm{Cu}}_{5}$ in parentheses).

**TABLE 3:** Band origins and vibrational frequencies observed in the photoelectron spectra in Figure 3.

Band	Origin	Peak	Frequency
A	3.143(10) eV		
		a	154(6) cm^−1^
B	3.650(8) eV		
		a	88(4) cm^−1^
		b	174(8) cm^−1^
		c	251(12) cm^−1^
		d	345(16) cm^−1^

In Figure 1, in the spectrum recorded at 325 nm two lower lying bands are visible, which correspond to electronically excited states of the neutral ${\mathrm{Cu}}_{5}$. In Figure 3, these two bands are presented in more detail—the spectrum of band B is the same as shown in Figure 1 for 325 nm, and the spectrum of band A has been measured at 365 nm. Band A exhibits a relatively short vibrational progression, hinting at rather similar geometries of ${\mathrm{Cu}}_{5}^{-}$ in its ground state and neutral ${\mathrm{Cu}}_{5}$ in its first excited state. It is dominated by one vibrational frequency (154 ${\mathrm{cm}}^{-1}$), but several modes with somewhat larger frequencies seem to contribute, as no vibrational peaks beyond peak 'a' can be resolved. Band B exhibits a broader vibrational progression, which indicates that the geometry of the second excited state of neutral ${\mathrm{Cu}}_{5}$ differs more from that of ${\mathrm{Cu}}_{5}^{-}$ than that of the first excited state. The four observed vibrational peaks are practically equidistant (Table 3), which could hint at the excitation of a single excited vibrational mode. The strong intensity modulation of the progression in this case would require a rather unusual Franck–Condon profile; it is therefore more probable that here two modes are dominantly excited, one with a frequency of 88 cm^−1^ and one with a frequency of 251 cm^−1^.

**FIGURE 3:**
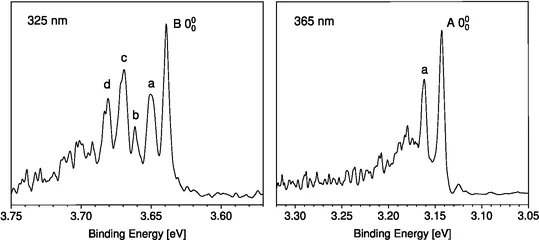
Photoelectron spectra of the two lower lying bands introduced in Figure 1, recorded at two different wavelengths.

### 3.1 Anisotropy

The angular distribution of photoelectrons emitted from randomly oriented atoms or molecules by absorption of a single photon of linearly polarised light can be described by

(1) $$\frac{d\sigma }{d\Omega }=\frac{\sigma_{\mathrm{tot}}}{4\pi }\left(1+\beta \left(\frac{3}{2}\cos^{2}\;\theta -\frac{1}{2}\right)\right)$$

where β is the anisotropy parameter and θ the angle with respect to the laser polarisation. For spherical systems an expression for β was derived by Bethe and extended by Cooper and Zare [25, 26]:

(2) $$\beta =\frac{l(l-1)R_{-}^{2}+(l+1)(l+2)R_{+}^{2}-6l(l+1)R_{-}R_{+}\cos\;\Delta \delta_{l}}{(2l+1)\left(lR_{-}^{2}+(l+1)R_{+}^{2}\right)}$$

It describes the interference between the two partial continuum waves with angular momenta of l±1 produced by emission from an initial state with angular momentum l. Here, $R_{\pm }=\int_{0}^{\infty }r^{3}R_{nl}\left(r\right)R_{kl\pm 1}\left(r\right)dr$ is the radial dipole matrix element connecting the initial and final state and $\Delta \delta_{l}$ is the phase difference between the two partial waves. As both the radial dipole matrix elements and the phase shift depend on the energy (or momentum) of the outgoing electron, β exhibits a pronounced energy dependence, except for s states where only an outgoing p wave is allowed.

A universal description of the dependence of β on the photoelectron momentum has recently been developed for spherical simple metal clusters [19]. It was shown that the curve β(k) exhibits a pronounced minimum close to k=l/R, where R is the cluster radius, and l is the initial angular momentum of the photoelectron (the image charge interaction shifts the minimum to a value slightly below l/R).

We have measured angle resolved spectra of ${\mathrm{Cu}}_{5}^{-}$ for a broad range of photon energies. As the momentum of the photoelectron is determined both by the photon energy and the final vibrational excitation of the cluster, we have first evaluated just the angular distributions of the 0–0 transition. In Figure ‍4, the fitted values of β are shown as a function of the reduced momentum kR. For simplicity we assumed the cluster to be spherical and calculated its radius as $R=r_{s}N^{1/3}=2.41Å$, using the bulk Wigner–Seitz radius $r_{s}=1.41Å$. In Figure 4, the theoretical curve β(kR) is shown, calculated for the same value of R and for l=1, the dominant angular momentum character of the HOMO (results for other radii are shown in the Supporting Information). The β‐curve was scaled by a factor of 0.7 to better fit the experimental data; such a reduction in interference contrast could be due to the deviation of the cluster shape from a perfect sphere. One can see that the experimental β values follow the prediction rather well, which is quite remarkable given that the theory was developed for larger clusters and larger angular momenta. Such a dependence on electron momentum and especially such a clear minimum in the β values has not been observed in earlier measurements on ${\mathrm{Na}}_{5}^{-}$‍ [27], which has the same structure as ${\mathrm{Cu}}_{5}^{-}$ and the same overall shape of the HOMO orbital, but in that study the measurements were just not performed at photon energies sufficiently close to threshold (that is at sufficiently small values of the electron momentum *k*). This calls for improved measurements on ${\mathrm{Na}}_{5}^{-}$, which would allow a direct comparison of the two systems.

**FIGURE 4:**
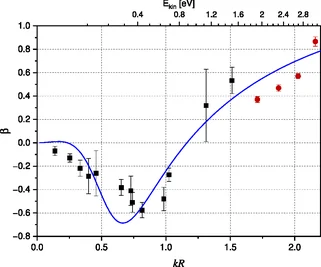
Anisotropy parameter β of the ${\text{Cu}}_{5}^{-}$ HOMO versus scaled electron momentum kR. The upper axis indicates the corresponding kinetic energy of the photoelectrons. Since β‐parameters differ for different vibrational states, β is shown for the 0–0 transition (for the highest kinetic energies, indicated by red spheres, the vibrations could not be resolved—here vibration‐averaged values are shown). The theoretical prediction of emission from a pure p state [19] is scaled by 0.7 in amplitude to match the experimental range.

It is now an interesting question whether the lowering of the kinetic energy of the outgoing electron due to vibrational excitation of the neutral cluster has the same effect on the angular distributions as a lowering caused by smaller photon energies.

In Figure 5, the values of β are depicted for all resolved peaks in the photoelectron spectra, that is for different amounts of vibrational excitation. One can see that in most cases the β‐values follow the overall behaviour of the 0–0 transitions, as shown in Figure 4. Such a dependence just on the final photoelectron energy has already been observed by Wang and coworkers, for the case of ${\mathrm{Au}}_{4}^{-}$ [28]. They compared β‐values for photoelectrons emitted at different photon energies and without vibrational excitation with those of photoelectrons emitted at one photon energy but with different amounts of vibrational excitation—the results were very similar. In our results there are exceptions, though; some β‐values deviate from the overall trend. This is most clearly seen for the cases of excitation at 480 and 490 nm; here the β‐values exhibit exactly the opposite trend than that seen for the 0–0 transitions, decreasing with increasing momentum. The deviation is most probably due to photoemission via an autodetaching excited state of the cluster anion, which leads to a more complex interplay between vibrational excitation and photoelectron angular distribution. This might also be the case for the excitation at 610 and 620 nm, where the β values exhibit a complex pattern. Similarly strong dependencies of angular distributions on vibrational excitation have already been observed for other systems, like ${\mathrm{AgF}}^{-}$ [29] or antracene anions [30]. A theoretical prediction of angular distributions in such cases would be highly interesting but certainly also constitutes a significant challenge.

**FIGURE 5:**
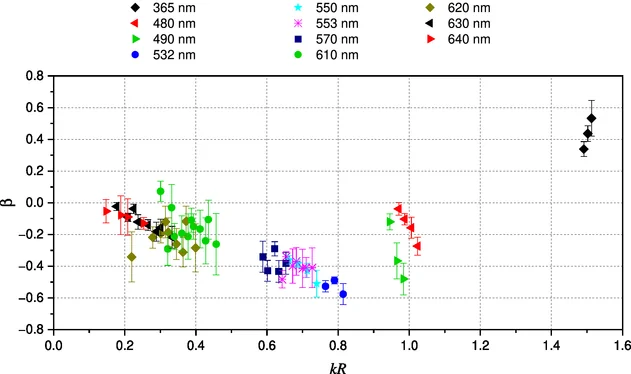
Vibrationally resolved anisotropy parameter β of the ${\text{Cu}}_{5}^{-}$ HOMO versus scaled electron momentum.

## 4 Conclusions

Angle‐resolved photoelectron spectra of ${\mathrm{Cu}}_{5}^{-}$ have been measured over a wide range of photon energies. The resolved vibrational progression yields a highly accurate adiabatic electron affinity value and values for the vibrational frequencies of the neutral cluster. The anisotropy parameter β for detachment from the HOMO follows the universal description developed for spherical clusters. In addition, a non‐trivial dependence of β on the vibrational excitation is observed for some photon energies, revealing a richer coupling between nuclear motion and angular distributions, probably caused by electron emission via autodetaching excited states. The results provide a stringent benchmark test for theory and open new avenues for studying electron emission dynamics in small metal clusters.

## Funding

This work was supported by the Deutsche Forschungsgemeinschaft (186940802, 443252596).

## Conflicts of Interest

The authors declare no conflict of interest.

## Supporting information

Supplementary Material

## Data Availability

The data that support the findings of this study are available from the corresponding author upon reasonable request.
