# Supplementary material for: High‐Resolution Angle‐Resolved Photoelectron Spectroscopy of Cu5− Clusters
Source: Chemphyschem. 2026 Oct 25;27(20):e70605. doi: 10.1002/cphc.70605 (PMC13696353; doi:10.1002/cphc.70605)
Supplement: Supplementary file 1 [file CPHC-27-e70605-s001.pdf]

Supporting Information:
High Resolution Angle-Resolved Photoelectron Spectroscopy of Cu_5^- Clusters

Lukas Weise, Steve Takouan Tchounga, and Bernd von Issendorff

Physikalisches Institut and Freiburg Material Research Center,
Universität Freiburg, Hermann-Herder-Str. 3, D-79104 Freiburg

Email: bernd.von.issendorff@uni-freiburg.de

DFT calculations

Calculations have been performed using two DFT-programs: Turbomole 7.41 [1] and Orca 6.11 [2]. In both cases the resolution of identity approximation has been employed (keyword “RI-J” in Turbomole, “RIJCOSX” in Orca).
The frequencies of the totally symmetric vibrational modes of the neutral cluster as given in the main text have been calculated using Turbomole at the PBE0/def2-TZVP level, after geometry optimization at the same level.

In order to calculate electron affinities for a given functional, the geometry of the anion and the neutral cluster have been optimized for this selected functional. Additionally, the single point energy of a neutral cluster with the optimized anionic geometry was calculated. The vertical detachment energy (VDE) is then taken as the difference of the total energy of the optimized anionic and the fixed geometry neutral clusters, the adiabatic detachment energy (ADE) as the difference of the total energies of optimized anionic and neutral clusters. The results are given below. The calculated electron affinities exhibit a strong dependence on the functional used and in most cases deviate significantly from the measured adiabatic electron affinity of 1.8947(12). Interestingly hybrid functionals like PBE0 or M06 do not perform better than functionals without exact exchange, nor does the range-separated functional wB97M-V.
For the popular PBE0 functional it was tested whether a larger basis set or the inclusion of dispersion interaction (which potentially could change the optimized geometry) can improve the result, but this was not the case. Of all functionals tested PBE performed best, but this might be slightly fortuitous, as an improvement of the basis set worsened the agreement with experiment.
It should be noted that for the B3P86 functional in combination with the lanl2dz basis set we obtained results different from those calculated by Li et al. using the GAUSSIAN09 package [3]. In combination with the def2-TZVP basis set this functional in fact performed almost as well as PBE.

Turbomole

Functional	Basis set	VDE [eV]	ADE [eV]
BP86	def2-TZVP	-2,058	-2,026
PBE	def2-TZVP	-1,929	-1,899
PBE	def2-TZVPPD	-1,967	-1,940
TPSS	def2-TZVP	-1,758	-1,730
PBE0	def2-TZVP	-1,641	-1,593
PBE0	def2-TZVPP	-1,655	-1,610
PBE0-D3	def2-TZVP	-1,642	-1,596

Orca

Functional	Basis set	VDE [eV]	ADE [eV]
BP86	def2-TZVP	-2,063	-2,030
PBE	def2-TZVP	-1,931	-1,899
PBE0	def2-TZVP	-1,637	-1,595
TPSS	def2-TZVP	-1,759	-1,730
B3P86	def2-TZVP	-1,874	-1,829
B3P86	lanl2dz	-1,798	-1,748
M06-L	def2-TZVP	-1,783	-1,755
M06	def2-TZVP	-2,042	-2,005
wB97M-V	def2-TZVPD	-1.579	-1.524

Universal angular distribution functions

The β-parameter for electron emission from a p-orbital as a function of the scaled electron momentum was calculated following the procedure published before [4]. The parameters for the interaction potential were a depth of the potential of $V_0 = -6.9$ eV and a slope of a = 1.3 Å (as derived in the Supplementary Material of [4]). We calculated the β-function for the radius of a spherical cluster with the volume of five copper atoms ($R = 2.41$ Å $= 4.56\ a_0$) as well as for smaller and larger radii. The results are shown below. One can observe that the angular distributions are close to independent of the cluster radius.

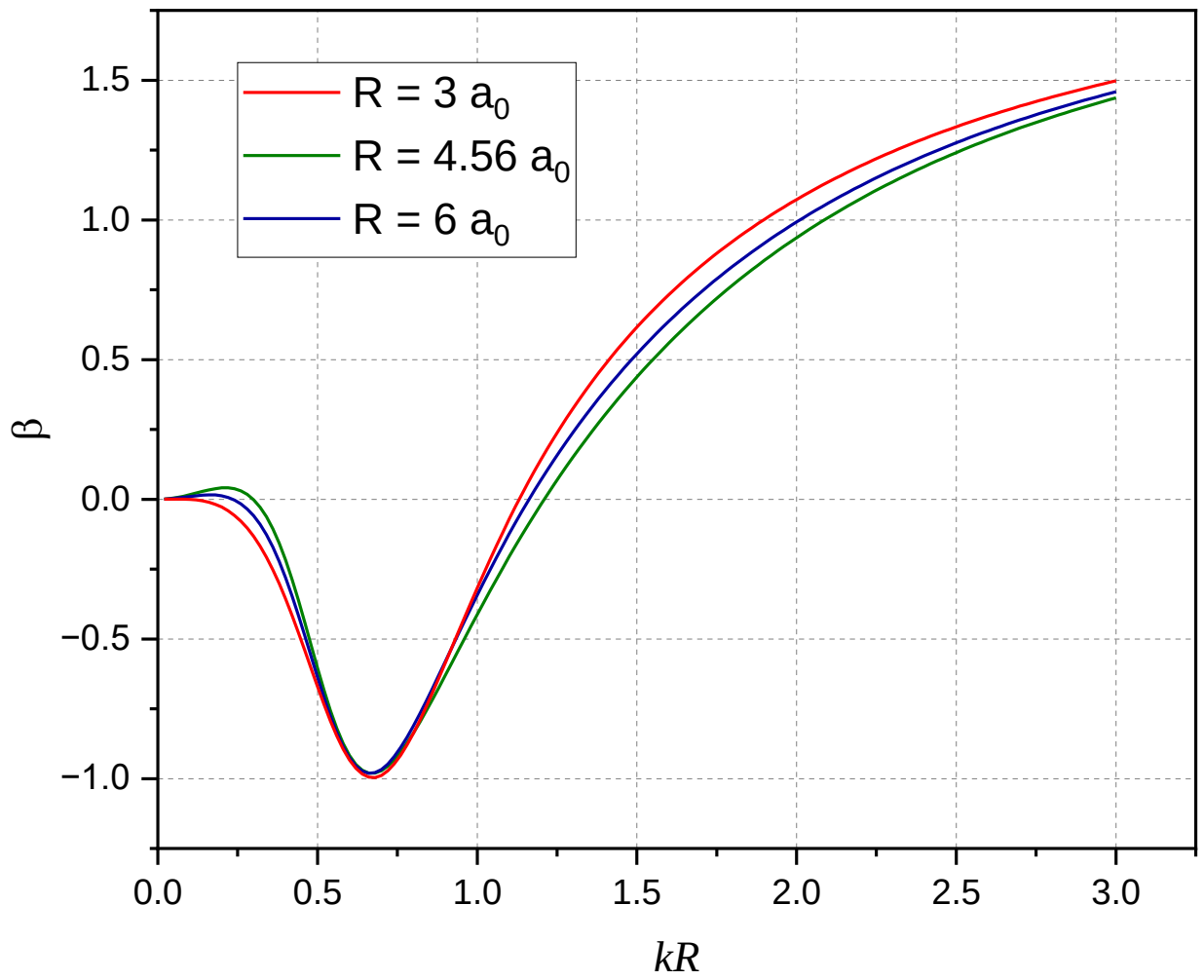

Figure S1: β-parameters for emission from a p-orbital for different copper cluster radii.

References

[1] TURBOMOLE V7.4.1 2019, a development of Universityof Karlsruhe and Forschungszentrum Karlsruhe GmbH, 1989-2007, TURBOMOLE GmbH, since 2007; available from https://www.turbomole.org.

[2] Neese, F. *Software update: the ORCA program system, version 6.0*, WIRES Comput. Molec. Sci. **15**, e70019 (2025), doi.org/10.1002/wcms.70019

[3] C.-G. Li, Z.-G. Shen, Y.-F. Hu, Y.-N. Tang, W.-G. Chen, and B.-Z. Ren, *Insights into the structures and electronic properties of* Cu_{n+1}^{μ} *and* Cu_nS^{μ} *(n = 1-12; μ = 0, ±1) clusters*, Sci. Rep. **7**, 1345 (2017)

[4] A. Piechaczek, C. Bartels, C. Hock, J.-M. Rost, and B. von Issendorff, *Decoherence-Induced Universality in Simple Metal Cluster Photoelectron Angular Distributions,* Phys. Rev. Lett. **126,** 233201 (2021)
